# Sustainable Hydrates for Enhanced Carbon Dioxide Capture from an Integrated Gasification Combined Cycle in a Fixed Bed Reactor

**DOI:** 10.1021/acs.iecr.1c01174

**Published:** 2021-07-15

**Authors:** Mohd Hafiz Abu Hassan, Farooq Sher, Bilal Fareed, Usman Ali, Ayesha Zafar, Muhammad Bilal, Hafiz M.N. Iqbal

**Affiliations:** † Fakulti Sains dan Teknologi, Universiti Sains Islam Malaysia, Bandar Baru Nilai, 71800 Nilai, Negeri Sembilan, Malaysia; ‡ Department of Engineering, School of Science and Technology, Nottingham Trent University, Nottingham NG11 8NS, U.K.; § Key Laboratory for Green Chemical Technology of Ministry of Education, School of Chemical Engineering and Technology, Tianjin University, Tianjin 300072, China; ∥ International Society of Engineering Science and Technology, Coventry CV1 5FB, U.K.; ⊥ Department of Chemical Engineering, University of Engineering and Technology, Lahore 54890, Pakistan; # Institute of Biochemistry and Biotechnology, Faculty of Biosciences, University of Veterinary and Animal Sciences, Lahore 54000, Pakistan; ¶ School of Life Science and Food Engineering, Huaiyin Institute of Technology, Huaian 223003, China; ∇ Tecnologico de Monterrey, School of Engineering and Sciences, Monterrey 64849, Mexico

## Abstract

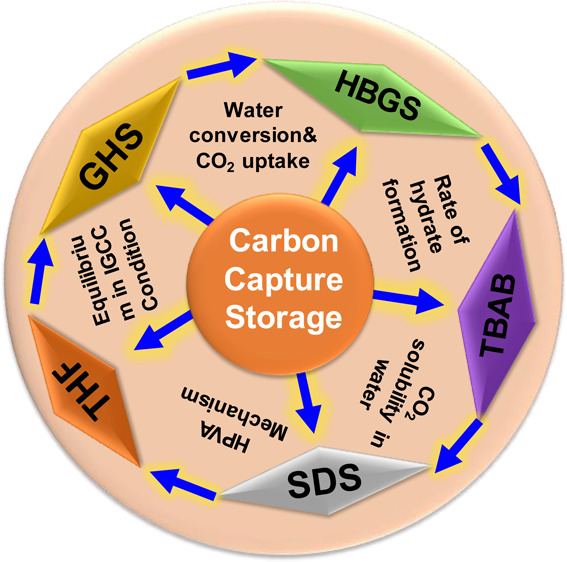

An increase in temperature of up to 2 °C occurs when the amount of CO_2_ reaches a range of 450 ppm. The permanent use of mineral oil is closely related to CO_2_ emissions. Maintaining the sustainability of fossil fuels and eliminating and reducing CO_2_ emissions is possible through carbon capture and storage (CCS) processes. One of the best ways to maintain CCS is hydrate-based gas separation. Selected type T1-5 (0.01 mol % sodium dodecyl sulphate (SDS) + 5.60 mol % tetrahydrofuran (THF), with the help of this silica gel promotion was strongly stimulated. A pressure of 36.5 bar of CO_2_ is needed in H_2_O to investigate the CO_2_ hydrate formation. Therefore, ethylene glycol monoethyl ether (EGME at 0.10 mol %) along with SDS (0.01 mol %) labeled as T1A-2 was used as an alternative to THF at the comparable working parameters in which CO_2_ uptake of 5.45 mmol of CO_2_/g of H_2_O was obtained. Additionally, it was found that with an increase in tetra-*n*-butyl ammonium bromide (TBAB) supplementation of CO_2_, the hydrate and operating capacity of the process increased. When the bed height was reduced from 3 cm to 2 cm with 0.1 mol % TBAB and 0.01% SDS (labelled as T3-2) in fixed bed reactor (FBR), the outcomes demonstrated a slight expansion in gas supply to 1.54 mmol of CO_2_/g of H_2_O at working states of 283 K and 70 bar. The gas selectivity experiment by using the high-pressure volume analysis through hydrate formation was performed in which the highest CO_2_ uptake for the employment of silica contacts with water in fuel gas mixture was observed in the non-IGCC conditions. Thus, two types of reactor configurations are being proposed for changing the process from batch to continuous with the employment of macroporous silica contacts with new consolidated promoters to improve the formation of CO_2_ hydrate in the IGCC conditions. Later, much work should be possible on this with an assortment of promoters and specific performance parameters. It was reported in previous work that the repeatability of equilibrium moisture content and gas uptake attained for the sample prepared by the highest rates of stirring was the greatest with the CIs of ±0.34 wt % and ±0.19 mmol of CO_2_/g of H_2_O respectively. This was due to the amount of water occluded inside silica gel pores was not an issue or in other words, vigorous stirring increased the spreadability. The variation of pore size to improve the process can be considered for future work.

## 1 Introduction

Gases are entrained through the effects of greenhouse gases, particularly, carbon dioxide (CO_2_) is found in the atmosphere and some of them return to earth. The increase in global warming is due to CO_2_ emission that is produced by burning coal, natural gas, and oil to meet industrial needs. A serious issue for global warming in this scenario is the anthropogenic emission of CO_2_ into the atmosphere.^1^ Tackling CO_2_ emission in a large scale is possible by using the carbon capture and storage (CCS) technique, and this is considered to be a promising technology. The dual challenge for CCS is to minimize the CO_2_ emissions and to meet the future energy requirements by employing fossil fuel reserves. Around the world, CCS is a very versatile technology and has recently received much attention to reduce global warming; it is expected that in 2050, CO_2_ emissions will reduce up to 90%.

The effort in reducing global warming has been captivated in the last few decades by using various methods, and all renewable technologies are essential. CCS is one of the most important processes for capturing and storing carbon in chemical plants and power stations. The installation of CCS will ensure that captured CO_2_ from various sources will be transported through a pipeline network into the deep ocean floor.

Oxides^2,3^ (ZnO and Fe_2_O_3_), hybrid oxides-amine,^4^ (CuO and MgO), metal–organic frameworks,^5^ and carbonaceous adsorbents^6,7^ have been continuously used in carbon capture technology. One of the carbon storage methods is the hydrate composition used to emit CO_2_,^8^ that is, 1.2 mmol of CO_2_/g for H_2_O in the gas phase, using an agitator that moves the center of the tank to get more CO_2_ hydrate formation at an operating condition of 80 bar and a temperature of 275 K.

Additionally, a lot of related research has been done in the past using physical and chemical CO_2_ imaging, zeolites, rare nanocomposites, chemical combustion, chemical gasification of integrated circulation (IGCC), advertising media, disinfected membranes, cryogenic polymers, and hydrate-based gas separation (HBGS).^9^ HBGS is one of the most energy-efficient, inexpensive, and encouraging methodologies in CO_2_ capture field.^9^ This CO_2_ capture strategy includes clathrate or hydrate crystallization and can be used in all transport (from a pipeline) and preignition (from petroleum gas).^10,11^ A proper ratio of water is needed to produce crystalline structures. The cycle depends on the volume of water to frame nonstoichiometric glasslike crystalline inside the grouping of CO_2_, N_2_, O_2_, and H_2_ as parts of combustible gases at high fixations (bar 10–70) and low temperatures (near 273 K).^12^ This strategy for isolation is acknowledged to be successful when the HBGS compelling at IGCC is just 4.4–8%^13,14^ and the energy consumption is less than 2.05 MJ/kg-CO_2_.

The HBGS cycle is more sensible in the precombustion of CO_2_ from a gas–gas compound than in a flue gas compound because the deficient pressing factor of the shipped gas is a few times higher than that of the oil gas in the post combustion capture. Therefore, the restriction of implementing the HBGS cycle in postcombustion will be the pressure costs and the requirement to construct huge CO_2_ capture equipment.^15^ Mainly, there are three conditions necessary for hydrate formation to happen: (i) low temperature and high pressure are required to form pure water depending upon the physical and chemical properties of the guest atom; (ii) particles should be obtainable, for example, methane, ethane, or CO_2_; (iii) an adequate measure of water used for the CO_2_ hydrate development method should be at the level of 272.15–282 K. This is the state of the CO_2_ precombustion framework. With the precombustion of CO_2_, the elimination of CO_2_ from the particles of gas can be done due to the large differences in the hydrate phase equilibrium of CO_2_ and H_2_.^16^ The initial pressure needed to form CO_2_ and H_2_ hydrates was around 20 and 5000 bar individually.^11^

As of now, there is the continuous use of utilizing oxygenated solvents to reduce the operating pressure, diminish the acceptance time, and accelerate the development rate. Oxygenated solvent is characterized as a natural solvent containing oxygen as a component of the atomic structure, for example, alcohols and ketones (EPA, 1970). Most recognized oxygenated solvent for HBGS is tetrahydrofuran (THF). THF capacity gradually decreases to form the capability of THF to be easily involved in the hydrate phase to produce the hydrate components. In addition, components of hydrate can also be supported by THF, wherein the THF effect on separation performance is related to the feed gas components. Pure CO_2_ gas consumption creates a big impact on hydrate formation. Sodium dodecyl sulphate (SDS)–water arrangement was made at various concentrations (500 ppm, 2000 ppm, and 4000 ppm). The experiments were performed at 274 K and 36 bar, and the SDS concentration of 4000 ppm showed very high water to hydrate conversion (52 mol %), which was 2 mol % more than a result obtained by pore filled silica with water. Likewise, the amount of gas uptake was more projecting than in the control experiment.

The effect of the surfactant on hydrate formation behavior was examined by using CO_2_ gas supply with a purity of more than 99%. Different surfactants were used as promoters in the past like SDS and tetra-*n*-butyl ammonium bromide (TBAB). Despite using these promoters, limited gaseous solubility and time taken for the process of hydrate formation are the major challenges for successful carbon capture applications.^17^ The effect of the surfactant on the performance of hydrate formation through CO_2_ gas supply with more than 99% purity was studied. It was reported that anionic surfactants such as SDS were better at improving water-repellent levels than cationic and nonionic surfactants. In TBAB hydrate, bromine is part of the cage structure, and the TBAB located at the center of four cages as a guest makes this semiclathrate hydrate stable even at atmospheric pressure and also easy to handle. When 0.3 mol % TBAB was employed in stirred tank reactor (STR), a 20% enhancement in gas uptake was observed compared to the most traditional method.^18^ They found that the addition of TBAB-enhanced CO_2_ engaged in the hydrate, and the driving force of the process was also increased when the operating conditions were shifted to 50 bar and 278 K.

However, the number of moles of CO_2_ transferred into the hydrate slurry phase decreased with the increase of TBAB concentration above 0.3 mol %. The hydrate phase equation in the fusion of fuel gas mixture and TBAB supplement in the range 283–290 K in the IGCC process with a pressure range of 25–50 bar was also studied.^19^ They pointed out (according to Raman’s analysis) that only CO_2_ molecules were merged under these experimental conditions which served as the basis for CO_2_ capture in the fuel gas mixture.^20,21^ Although it has been suggested that the hydrating process may be used in the IGCC process without significantly lowering the processing temperature, reversing the use of this method was a condition of force to mix the mixture of water additives. Therefore, the employment performance of 0.3 mol % TBAB in fixed bed reactor (FBR) was investigated, and the results showed a slight increase in gas uptake to 1.2 mmol of CO_2_/g of H_2_O in the operating conditions of 279 K and 60 bar.^22,23^ Furthermore, it was observed that as long as SDS is used, clathrate hydrate is formed naturally by increasing the contact area between the gas and water phase; while THF can be positively enhanced by SDS, it would make sense to see THF hydrate crystals alleviate the system and act as a CO_2_ hydrate catalyst.^24,25^

The main aim of the present study is to capture CO_2_ after the formation of hydrate using ethylene glycol monoethyl ether (EGME) and TBAB in a fixed bed reactor. Solid adsorbent known as silica gel was used in this work helped to eradicate the stirring phenomena within the reactor. EGME and TBAB were selected to increase the operating capacity of the process. The fuel gas mixture (40% CO_2_ and 60% H_2_) was processed by high pressure volume analysis (HPVA). Furthermore, oxygen-solved solvents are helpful for hydrate formation; especially, mono ethylene ether of ethylene glycol has been identified as a potential replacement for THF.

This CO_2_ capture method involves clathrate or gas hydrate crystallization and can be applied to both post- (from flue gas) and precombustion (from fuel gas) capture, respectively. The process relies on the ability of water to form nonstoichiometric crystalline compounds in the presence of CO_2_, N_2_, O_2_, and H_2_ as well as natural gas components at high pressures (10–70 bar) and low temperatures (near 273 K). This separation method is believed to be energy efficient, where the energy penalty imposed by HBGS in the IGCC is only 4.4–8.0% and the energy consumption could be as low as 2.05 MJ/kg-CO_2_.^26^ Moreover, hydrate additives such as THF and SDS can be used to reduce the formation components of hydrate to enhance the hydrate rate and improve the differentiation performance.

## 2 Experimental Section

### 2.1 Chemicals

Oxolane or THF, an organic compound with the chemical formula (CH_2_)_4_O, EGME, SDS, and additives of TBAB with a purity of 99.71, 99.50, 99.60, and 97.80%, respectively, were used. Silica gel with a pore volume of 0.630 cm^3^/g, an area of more than 499 m^2^/g, a standard molecule size of 200–500 μm, and a pore size of 5.14 nm was used. All synthetic substances were bought from Acros Organics. He and N_2_ gas were used for cleaning and controlling the high pressing factor volumetric analyzer valve. Antifreeze fluid was purchased from ASDA.

### 2.2 Preparation of Sample

Four methods were used to prepare the saturated silica gel. Method 1 contains naturally saturated silica that adsorbed moisture, method 2 contains very low stirring level of silica, method 3 describes the engagement of silica in bulk water, and method 4 contains the highest stirring level of silica. Method 4 was reported to give the highest CO_2_ uptake.^27^ As for this work, at the start of each test, silica gel was put into the oven and dried for one night. The used oven (model AX30) had the highest temperature of 250 °C and the lowest temperature of 40 °C. By all means, the final weight of each wet silica gel was subtracted from the dry silica gel weight to obtain the final moisture content. The weighing balance (model: AEA—220A) weighed up to a maximum weight of up to 220 g and a minimum weight of 10 mg. The promoters THF, and SDS were used, and dilution of the promoter was possible to attain THF of 3.00 mol %, TBAB of 0.29 mol %, and SDS of 0.01 mol %. The required amount of each promoter was added to 2.5 g of dry silica gel to prepare the sample. The quantity of each sample should be 2.5 g by using the dry silica gel.^28,29^

Additionally, various aqueous solutions of 95 g were prepared to produce a total amount of 100 g silica (5 g) combined with promoters. T1-5 sample was prepared by combining 5.60 mol % THF (9.11 g of THF) with 0.01 mol % SDS, (0.076 g of SDS) and 85.81 g of H_2_O. Next, T3-2 sample was prepared by combining 0.10 mol % TBAB (0.835 g of TBAB) with 0.01 mol % SDS (0.076 g of SDS) and 94.09 g of H_2_O. Finally, T1A-2 sample was prepared by combining 0.10 mol % EGME (0.237 g of EGME) with 0.01 mol % SDS (0.076 g of SDS) and 94.687 g of H_2_O. These samples were prepared using high stirring according to method 4.^27,30^

### 2.3 Experimental Procedure

**Figure 1:**
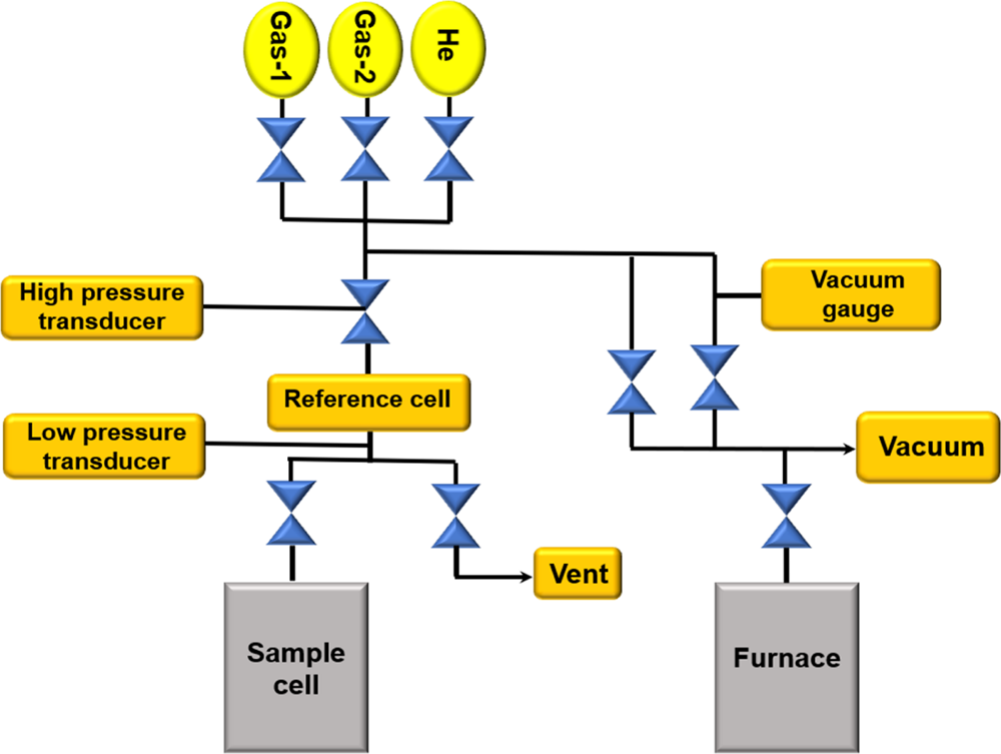
Schematic diagram of a high-pressure volumetric analyzer.

A schematic diagram of high-pressure volumetric analyzer is shown in Figure 1. Before the investigation, the framework was hand-cleaned for any impurities. This procedure was repeated multiple times, after which, the sample cell was filled with saturated silica gel and submerged in water bath. The cell valve was initially closed. After that, the cell was pressurized with a feeder vessel containing CO_2_ gas, and instantaneously, the ideal temperature was attained through constant temperature bath. Then, the cell valve was fully opened. From that point onward, the test stayed substantial for 1200 min. The pressure factor of the environment is limited during a similar temperature of the hydrate decay activity. From that point onward, the framework was naturally dumped with He gas a few times to clear the line.

## 3 Results and Discussion

### 3.1 Hydrate Formation Analysis

Generally, the observed rate of hydrate formation demonstrated that the use of promoters improved hydrate formation as reported by previous work.^27^ CO_2_ was released due to the preparation of hydrate, and it is possible because of the T1-3 effect. After the dynamic investigation of these three examples T1-1, T1-5, and T1-2, T1-1 had the highest active development in contrast to T1-5 and T1-2. The distinction between these three examples was comparable in terms of energy and was over 31% more prominent than other arranged examples, and the beginning energy at 0.09 mmol of CO_2_/g of H_2_O/min was comparable. Finally, T1-3 (0.06 mmol of CO_2_/g of H_2_O/min) showed more moderate energy among all T1 energies, which were moreover slower than the results from a single stimulant.

**Figure 2:**
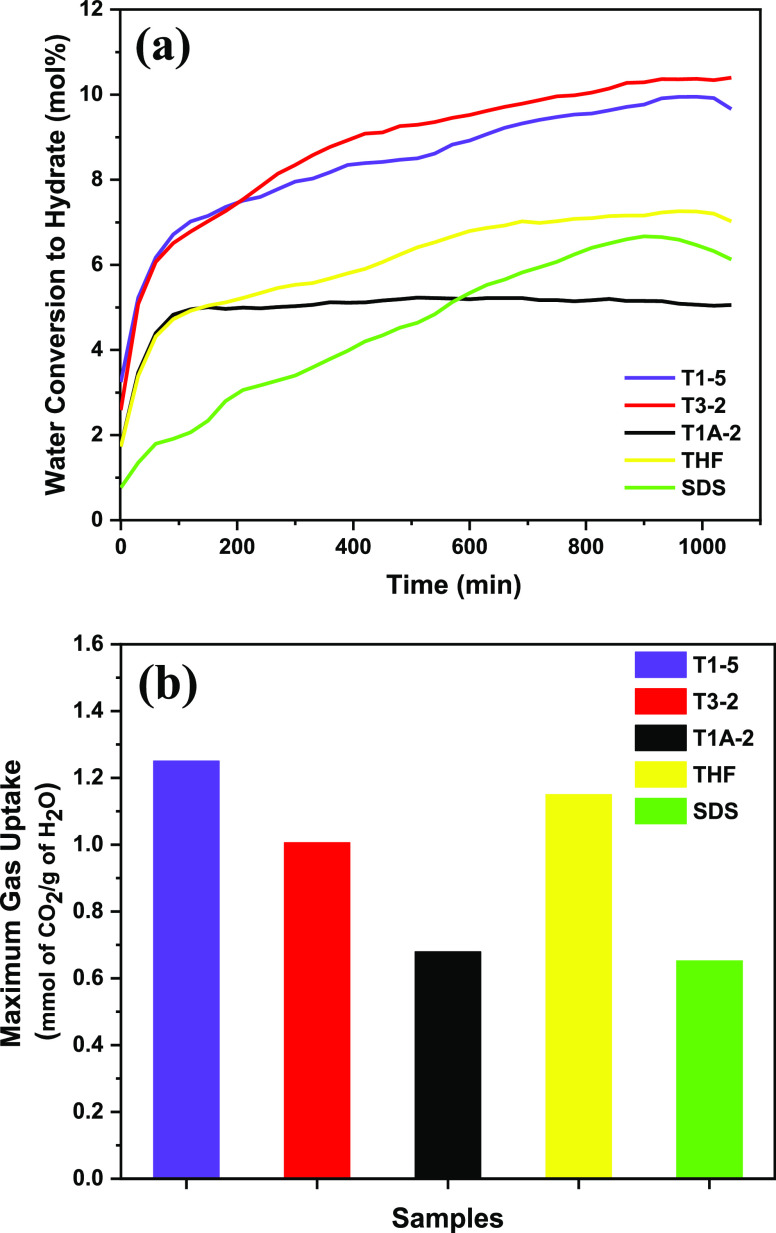
Comparison of (a) water conversion to hydrate and (b) gas uptake for T1-5, T3-2, T1A-2, THF (3.00 mol %), and SDS (0.01 mol %) at 288 K and 36 bar in 1200 min.

Considering these insights, it was assumed that THF in 3 mol % showed quick energy together with 0.01 mol % SDS, practically half and more prominent than a single dissolvable in a comparative zone. Hydrate formation and gas uptake of different samples (T1-5, T3-2, and T1A-2) are shown in Figure 2. SDS with 0.01 mol % had shown tremendous improvement in water conversion to hydrate and maximum CO_2_ uptake by almost 50% respectively after combined with 5.60 mol % THF (labeled as T1-5) as well as 0.10 mol % TBAB (labeled as T3-2). Assessment of gas uptake of various models of SDS (T1-5, and T3-2) are shown in Table 1. Consequently, it was confirmed that SDS at 0.01 mol % gave the highest amount of hydrate formation and CO_2_ uptake.^31^ Then, THF at 3 mol % referenced high active, and high gas dispersal was seen at 5.6 mol %. In the end, the hydrate improvement rate was not clearly comparative with high promoters’ concentration.

**Table 1:** Study of Gas Uptake at 36 bar and Working Temperatures of 288 and 293 K in 1200 minutes of Different Samples (T1-5, T3-2, and baseline experiments; SiG-H_2_O)

operating conditions	sample	exp. no.	number of moles of water (mmol)	CO_2_ formed in hydrate (mmol)	mean CO_2_ formed in hydrate (mmol)	CO_2_ uptake (mmol of CO_2_/g of H_2_O)	mean CO_2_ uptake (mmol of CO_2_/g of H_2_O) (90% CI)	SD
275 K & 36 bar	T1-5	1	3.70	0.39	0.40	5.82	5.95 ± 0.21	0.18
		2	3.70	0.41		6.08		
	T3-2	1	2.40	0.23	0.24	5.36	5.57 ± 0.34	0.29
		2	2.40	0.25		5.77		
	SiG-H_2_O	1	4.10	0.29	0.31	3.93	4.04 ± 0.17	0.15
		2	4.30	0.32		4.14		
275 K & 30 bar	T1-5	1	3.70	0.15	0.17	2.62	2.81 ± 0.30	0.26
		2	3.70	0.18		2.99		
	T3-2	1	2.40	0.13	0.14	3.09	3.28 ± 0.31	0.27
		2	2.40	0.15		3.47		
	SiG-H_2_O	1	4.20	0.14	0.13	1.82	1.71 ± 0.19	0.16
		2	4.10	0.12		1.60		
275 K & 22 bar	T1-5	1	3.70	0.05	0.05	0.91	0.86 ± 0.09	0.08
		2	3.70	0.04		0.80		
	T3-2	1	2.40	0.01	0.01	0.35	0.30 ± 0.09	0.08
		2	2.40	0.01		0.24		
	SiG-H_2_O	1	4.10	0.02	0.03	0.28	0.32 ± 0.06	0.05
		2	4.20	0.03		0.35		

### 3.2 Rate of Hydrate Formation Analysis

**Figure 3:**
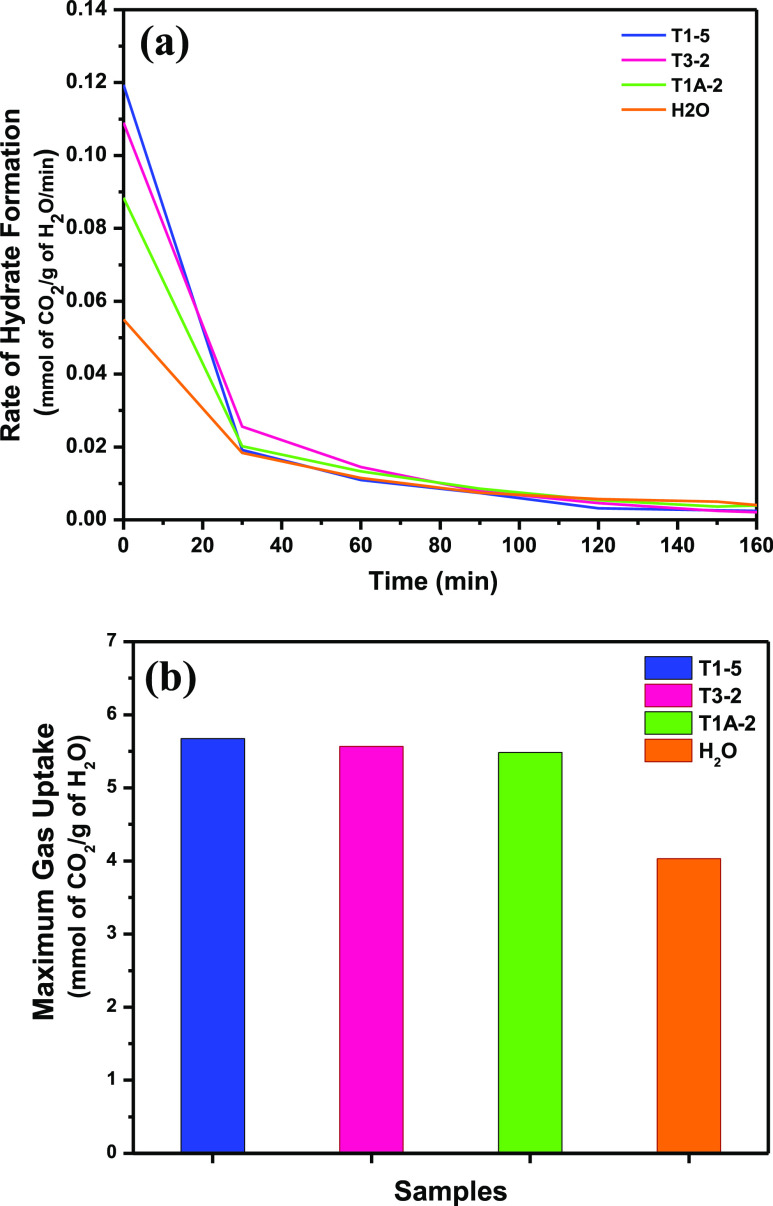
Study of (a) rate of hydrate formation for the ideal concentration of each combined promoter and baseline experiment at the working conditions of 275 K and 36 bar and (b) maximum CO_2_ uptake.

**Figure 4:**
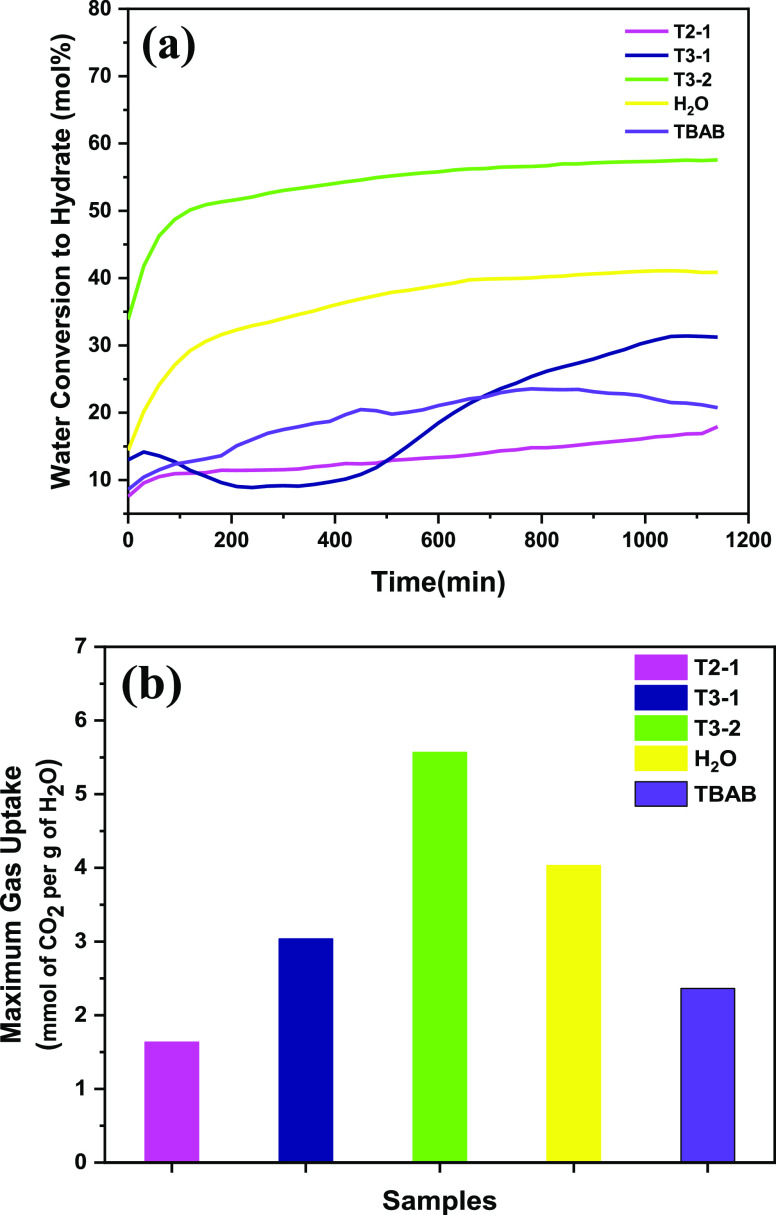
Correlation of (a) water transformation to hydrate and (b) maximum CO_2_ uptake for different T2 and T3 , TBAB at 0.29 mol%, and benchmark (SiG-H_2_O) tests at the working states of 275 K and 36 bar in 1200 minutes.

The rate of hydrate formation in various samples (T1-5, T3-2, T1A-2, and baseline experiment; SiG-H_2_O) were evaluated wherein the highest kinetic was obtained by T1-5 (0.01 mol % SDS and 5.60 mol % THF), followed by T3-2 (0.01 mol % SDS and 0.10 mol % TBAB), T1A-2 (0.01 mol % SDS and 0.10 mol % EGME) and baseline experiment (SiG-H_2_O) as shown in Figure 3 (a). The fundamental hydrate of T3-1 (0.01 mol % SDS and 0.29 mol % TBAB) at around 3 mmol of CO_2_/g of H_2_O every moment was half lower than T3-2 (Figure 4). T2-1 (3 mol % THF and 0.29 mol % TBAB) exhibited moderate dynamic energy, where no indication of progress in hydrate arrangement was clear. As per this perception, the occurrence of 3 mol % THF and 0.29 mol % TBAB inside silica gel pores was not ideal for hydrate arrangement, and low grouping of TBAB (0.1 mol %) was compulsory for hydrate development.^25,32^ In addition, the initial energy of T1A-2 (0.01 mol % SDS and 0.10 mol % EGME) was 0.09 mmol CO_2_/g of H_2_O/min and also quicker than benchmark test.

Medium EGME extraction (0.1 mol %) can expand hydrate development and high CO_2_ ingestion as demonstrated by T1A-2. The level of hydrate improvement at 275 K and 36 bar shows that 5.6 mol % THF to the structure of T1-5 has completely improved the result achieved by the baseline experiment from 0.06 to 0.12 mmol CO_2_/g of H_2_O/min as shown in Figure 3. Additionally, 0.1 mol % TBAB and 0.1 mol % EGME choices at 0.010 mol % SDS are similarly to fold the underlying hydrate rate improvement of baseline experiment as tended by T3-2 and T1A-2 independently. This has shown that the blending of different added substances with the SDS has redesigned the hydrate improvement, probably known as the synergic impact. The same improvement was observed for maximum gas uptake attained by T1-5, T3-2 and T1A-2 as depicted in Figure 3(b) in which the gas uptake had increased for almost 50% as compared to the baseline experiment.

### 3.3 Equilibrium Moisture Content at Different Combined Concentrations

Grouping of SDS fixed at 0.01 mol % with various concentrations of EGME expanded from 0.01 (T1A-1) to 1 mol % (T1A-3) had shown that the equilibrium moisture content relatively diminished from 12.89 ± 0.48 to 12.06 ± 0.46 wt %. These outcomes were contrasted, and the pattern test and clearly the presence of promoters inside silica gel pores decreased the measure of water accessible for hydrate formation. A similar pattern was likewise noticed for T1-5, and T3-2 blended promoters in which the existence of promoters showed a low equilibrium moisture content as compared to the baseline experiment (Table 1). The measure of water accessible inside silica gel pores within the sight of promoters did not promptly influence the greatest CO_2_ uptake and rate of hydrate formation. Along these lines, the investigations on different T1A tests were performed to contemplate the ideal grouping of EGME.

The outcomes sum up the equilibrium moisture content determined for all samples at specific concentrations. The presence of THF in T2-1 (0.29 mol % TBAB and 3 mol % THF) expanded the content to 10.25 ± 0.42 wt % from 8.48 wt % for a single TBAB supplement (0.29 mol %). In any case, the development of SDS totally decreased the measure of water inside the silica gel pores that were accessible for hydrate detailing. Low TBAB obsession in progressed improved moisture content by generally 0.1 wt % appeared differently in relation to T3-1 (0.01 mol % SDS and 1.00 mol % TBAB). The presence of THF fluid arrangement in TBAB may improve the moisture substance of the balance, which was not the circumstance with SDS. CI that saw T2 and T3 tests were higher than T1; anyway, the outcomes were till now considered revival.

The formation of hydrate is closely related to the amount of water available in the system. The total amount of the equilibrium moisture content was determined by subtracting the weight of wet silica gel with the weight of dry silica gel. It was observed that the sample prepared by vigorous stirring or also known as Method 4 had the highest moisture content with the lowest CI: 14.79 ± 0.34 wt %. The employment of vigorous stirring was expected to give this result which is essential for the CO_2_ hydrate formation process. Then, it was followed by Method 3 (silica was submerged in excess water) (13.83 ± 2.87 wt %), Method 2 (the lowest rates of stirring of silica) (13.64 ± 0.51 wt %) and Method 1 (silica was left to naturally adsorbed moisture) (13.56 ± 2.45 wt %). The relatively low CI obtained by Method 4 and Method 2 is explained by the need for stirring during sample preparation to ensure the water is well distributed inside silica gel pores. This also indicated that the samples prepared from both methods had high reproducibility.^27^

**Figure 5:**
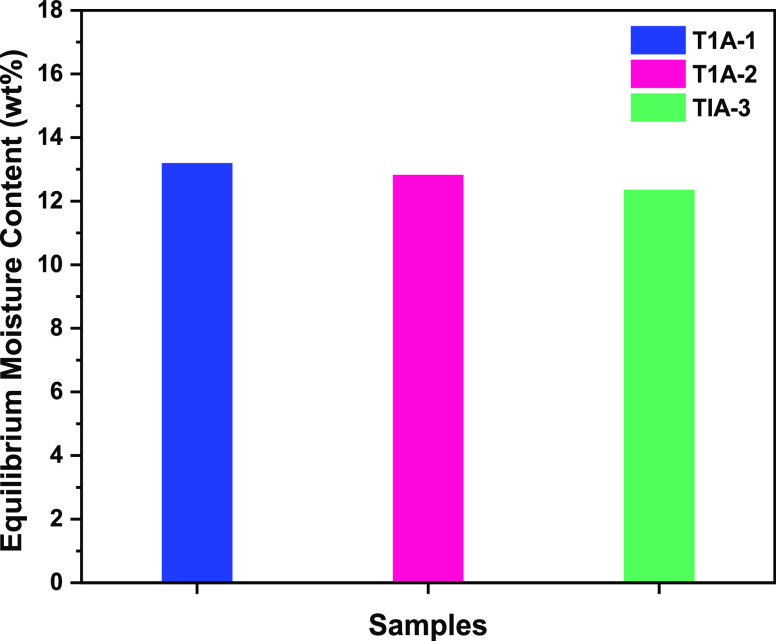
Summary of results for equilibrium moisture content at different samples of T1A-1, T1A-2, and T1A-3.

Hence, in this work the equilibrium moisture content inside silica gel pores increased from 12.06 ± 0.46 to 12.89 ± 0.48 wt % as the concentration of EGME was reduced from 1 (T1A-3) to 0.01 mol % (T1A-1) as shown in Figure 5 and Table 2. Additionally, the second lowest equilibrium moisture content was obtained at 0.1 mol % of EGME (T1A-2), which showed that the effect of promoters concentration toward the amount of water present inside the silica gel pores. Previously, it was reported that the highest equilibrium moisture content was achieved for T1-4 (14.36 ± 0.69 wt %), followed by T1-6 (13.90 ± 0.26 wt %), T1-1 (13.81 ± 0.14 wt %), T1-2 (13.71 ± 0.15 wt %), T1-5 (13.28 ± 0.18 wt %), T1-3 (13.17 ± 0.06 wt %), and T1-7 (13.14 ± 0.16 wt %).^27^ The CI observed was relatively low for all T1 samples which indicated that this combined promoter was well distributed inside the silica gel pores when vigorous stirring was employed during sample preparation.^33^

**Table 2:** Summary of Results for Equilibrium Moisture Content at Different Groupings of Type T1A-1, T1A-2, and T1A-3 Blended Promoters

	promoter concentration (mol %)					
sample	EGME	SDS	exp. no.	equilibrium moisture content (wt %)	mean equilibrium moisture content (wt %) (90% CI)	SD
T1A-1	0.01	0.01	1	13.18	12.89 ± 0.48	0.41
			2	12.60		
T1A-2	0.10	0.01	1	12.81	12.59 ± 0.36	0.31
			2	12.38		
T1A-3	1.00	0.01	1	11.78	12.06 ± 0.46	0.39
			2	12.34		

### 3.4 Water Conversion and CO_2_ Uptake through Hydrate Formation

**Figure 6:**
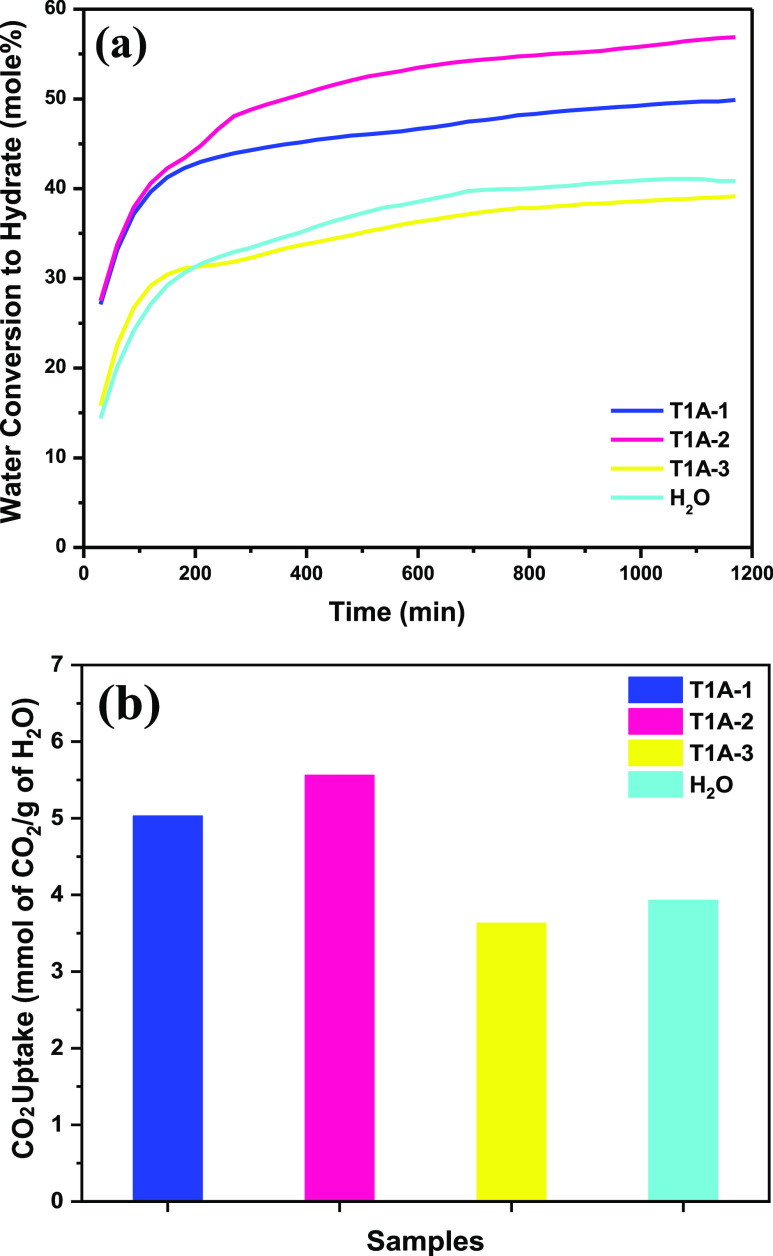
Comparison of (a) water transformation to hydrate and (b) greatest CO_2_ uptake for different T1A tests and standard investigation at the working condition of 275 K and 36 bar.

Hydrate formation tests were performed at 275 K and 35 bar in HPVA utilizing different samples arranged with around 0.5 g of saturated silica. The hydrate formulation cycle and formulas were utilized to calculate the transformation of water into hydrate of CO_2_. A hydration number of 5.75 was utilized to figure the transformation of water into hydrate as six water atoms are needed to frame CO_2_ hydrate (CO_2_·6H_2_O). The measure of water molecules determined in all T1A tests was roughly 3 mmol, used to compute the hydrate formation and CO_2_ uptake individually. A different grouping of various blended promoters is presented in Table 2. The outcomes acquired were looked at and the CO_2_ hydrate formation with EGME was seen the highest for TA1-2 with the assessment of more than 55 mol %, followed by T1A-1, which was 5 mol % lower than TA1-2. Be that as it may, T1A-3 indicated an inhibitory impact compared with baseline testing with just 35 mol % hydrate formation was observed as reported in Figure 6.

### 3.5 Occurrence of Hydrate from Water Conversion and CO_2_ Consumption

Mole quantity of H_2_O determined for T3-2 was approximately 2 mmol. The number of moles of water calculated for all T1A samples was around 3 mmol and was used to calculate the water conversion to hydrate and CO_2_ uptake. The number of moles of water for each sample is important to ascertain water change to hydrate and CO_2_ uptake individually. The outcomes obtained in this segment were compared with the baseline experiment, which was accounted for in the past section and silica reached to 0.29 mol % TBAB (Figure 4). The number of moles of water calculated for all T1A samples was around 3 mmol and was used to calculate the water conversion to hydrate and CO_2_ uptake. The highest water conversion to hydrate was observed for TA1-2 with a value of more than 55 mol %, followed by T1A-1 (0.01 mol % SDS and 0.01 mol % EGME) which was 5 mol % lower than TA1-2 (0.01 mol % SDS and 0.10 mol % EGME). However, T1A-3 (0.01 mol % SDS and 1.00 mol % EGME) showed an inhibition effect compared to the baseline experiment with only 35 mol % conversion. The same trend was observed for the maximum CO_2_ uptake as shown in Figure 6.^34−36^

The CO_2_ dissolved in water within the sight of hydrate has appeared, where the progress of hydrate was noticed. The water changed to hydrate for each sample. The expansion of THF at 3 mol % (T2-1; 3 mol % THF and 0.29 mol % TBAB) and SDS at 0.01 mol % (T3-1; 0.01 mol % SDS and 0.29 mol % TBAB) did not exclude the inhabitation impact which was recently shown by TBAB (0.29 mol %) reached with silica gel alone as depicted in Figure 4. The water change to hydrate obtained by the two blended samples (T2-1 and T3-1) was 75 and 25% lower when compared with the pattern analyzed for benchmark test (SiG-H_2_O). However, the water change accomplished by T3-1 had improved by almost 50% as compared to the product accomplished by 0.29 mol % TBAB. Consequently, it was normal that 3 mol % THF could not upgrade the rate of hydrate formation, although 0.01 mol % SDS could. Subsequently, T3-2 was set up in which the convergence of TBAB concentration was decreased to 0.1 mol % and SDS continued as before. Accordingly, the complete water change to hydrate improved definitely which was half higher (almost 60 mol %) than the baseline test (40 mol %).

The highest concentration of CO_2_ was achieved by T3-2 with a ratio of 5.57 ± 0.34 (Table 1). This was followed by T3-1 which was almost half lower than T3-2 and a half higher than T2-1.^37^ As the concentration of TBAB increases, the conversion of water to hydrate and excess CO_2_ absorption is reduced. In addition, the effect of inhibiting hydrate formation was observed. In this way, the positive concentration obtained by TBAB was 0.1 mol % in the 0.01 mol % SDS group. The CO_2_ dissolvability in water within the presence of hydrate was observed in which the development of hydrate was noticed for all T1A tests. The number of moles of water determined for all T1A tests was around 3 mmol and was utilized to figure the water transformation to hydrate and CO_2_ uptake separately. The results acquired in this section were contrasted and compared to the baseline experiment, as shown in Figure 6. The most elevated water change in hydrate was noticed in TA1-2 with an average of over 55% mol, followed by T1A-1. However, T1A-3 (1.00 mol % EGME S and 0.10 mol % SDS) indicated an inhibitory impact contrasted with baseline tests (40 mol %) regarding just 35 mol % water transformation to hydrate.

### 3.6 CO_2_ Solubility in Water

The CO_2_–H_2_ solubility in water has not yet been accounted for in the literature. The mole division of H_2_ gas break-up in the water is excessively little at the IGCC working pressures. The mole portion of CO_2_ disintegrated in the water at the partial pressure of CO_2_ in the fuel gas mixture was assumed to be equivalent to pure CO_2_ gas within the presence of hydrate. The introduced mole division of H_2_ break-up in the water at the IGCC conditions (*T* = 283 K) was found to be 0.0005 at 58 bar (*P*_H2_ = 35 bar and PCO_2_ = 23 bar) and 0.0006 at 70 bar (*P*_H_2__P = 42 bar and *P*_CO_2__P = 28 bar). These values are too small as relevant to the fraction of water at 283 K in pure CO_2_ gas with the value of 0.0175 at *P*_CO_2__P = 23 bar and 0.0185 at *P*_CO_2__P = 28 bar.

The initial study at 283 K and 58 bar and the bed stature of T1-5 and T3-2 did not show any hydrate formation in 1200 min. At that point, the bed height of T1-5 was decreased to 2 cm, yet the CO_2_ break-up in the water just marginally expanded to around 0.014 mole part of CO_2_ with no hydrate observed. Hence, a negligible formation of the hydrate was seen after 2500 min at these conditions when the long analysis was conducted. The driving force was then increased which resulted in a pressure bar operating at 70–283 K where various bed heights were used to investigate the hydrate formation in these operating conditions as shown in Table 3 in the next section.

**Table 3:** Correlation of Gas Uptake at Different IGCC Working Conditions and Bed Heights of T1-5 and T3-2 at a Temperature of 283 K and Pressure Factors of 58 and 70 bar.

sample	T1-5					T3-2				
operating conditions	283 K and 58 bar		283 K and 58 bar		283 K and 70 bar	283 K and 58 bar		283 K and 70 bar		283 K and 70 bar
bed height (cm)	3		2		2	3		3		2
experiment	1	2	1	2	1	1	2	1	2	1
number of moles of water (mmol)	3.9	3.8	2.3	2.3	2.3	2.4	2.4	2.4	2.4	1.4
CO_2_ formed in hydrate (mmol)				0.04	0.04					0.03
ratio of CO_2_ consumed per amount of water (mmol CO_2_ per mmol of H_2_O)				0.01	0.02					0.02
CO_2_ uptake (mmol of CO_2_/g of H_2_O)				1.08	1.11					1.54

### 3.7 Operating Conditions of IGCC

An assessment was performed between results achieved in this work and the past works for the IGCC conditions. Correlation of gas uptake at different conditions and bed statures of T1-5 and T3-2 are presented in Table 3. Utilizing mesoporous silica in batch FBR at the IGCC conditions are feasible. Also, the advantage of employing horizontal batch FBR as compared with the vertical setup is featured. The highest impact was accomplished by extracting 2.4 mmol of CO_2_/g of H_2_O utilizing 5.56 mol % THF and silica sand (macroporous silica) in bunch FBR at 283 K and 60 bar.^38^ This was followed by results accomplished in this assignment utilizing T3-2 (0.10 mol % TBAB and 0.01 mol % SDS) and T1-5 (0.01 mol % SDS and 5.60 mol % THF). A helpful impact was seen for T3-2 at various temperatures and pressures, where gas uptake attained was 1.548 mmol of CO_2_ per H_2_O g which was 40% higher than that for T1-5.

**Figure 7:**
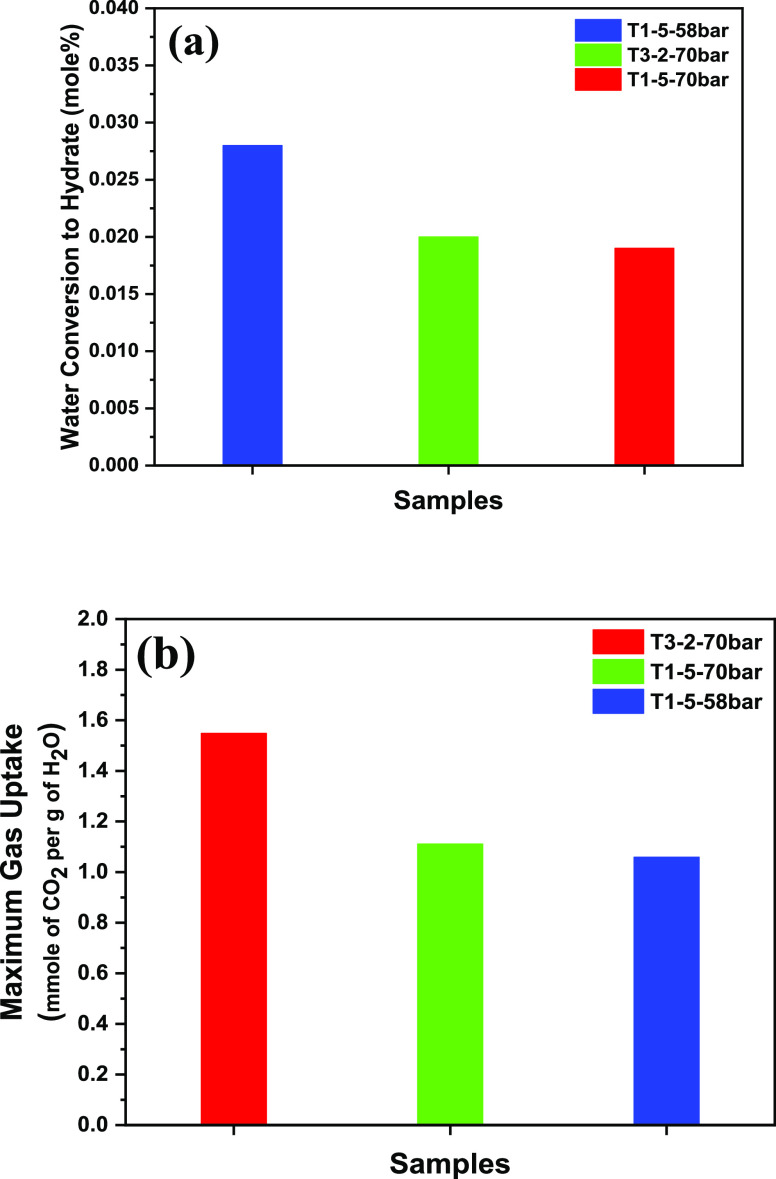
Correlation of (a) water transformation to hydrate and (b) gas uptake for T1-5 2 cm and T3-2 2 cm at 283 K and different IGCC working pressures.

Conversely, low gas admission was acquired at T1-5 at 283 K and 58 bar (long experiment) with a 0.461 mmol convergence of CO_2_/g of H_2_O contrasted to T3-2 at 283 K and 70 bar is because of contrasts in driving power. Water changed to the formation of hydrate and uptake of gas for T1-5,2 cm and T3-2,2 cm were occured at a reduced bed height, as shown in Figure 7. The gas mixture of gas conditions of IGCC conditions was required due to the mesoporous silica activity that altered the hydrate phase equilibrium in the inhibitory region. Similarly, Zheng et al. (2016) reported that their horizontal FBR batch means that the shortcut space was available compared to the straightforward setting. On the other hand, lower results in this work (with the highest CO_2_ uptake of 1.54 mmol of CO_2_/g of H_2_O) are expected due to the large internal area of the porous medium attained by horizontal FBR (2.4 mmol of CO_2_/g of H_2_O).

### 3.8 Hydrate Formation Mechanism in the HPVA

**Figure 8:**
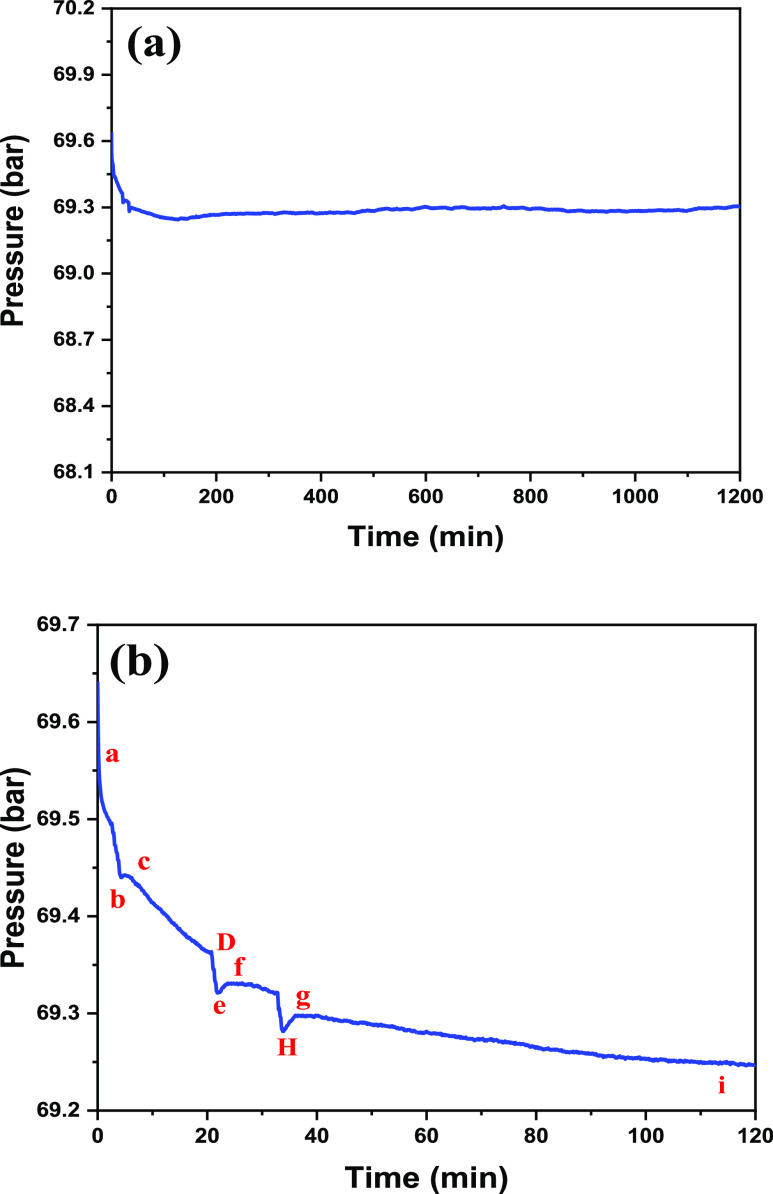
(a) *P*–*t* curve for T3-2 at 283 K and 70 bar in a fuel gas mixture system after 1200 min inside batch FBR (b) (inset is the *P*–*t* curve for the first 100 min; points D and H are the process repetition of points (b,c) and (e–g), respectively).

Fuel gas mixture system after 1200 min inside batch FBR is shown in Figure 8. As a result, the *P*–*t* curve for all assessments that indicated hydrate headway either in pure CO_2_ or fuel gas combination system was close to the model. *P*–*t* of all experiments that showed hydrate improvement either in pure CO_2_ or in a gas mixing system exhibited a comparative model. *P-t* curve of T3-2 at 283 K and 70 bar in fuel gas mixture following 1200 minutes inside the FBR gathering (embedded *P-t* twist of initial 100 minutes) such as in the cases of IGCC liked is presented in detail. Few phases of pressure drop referenced that there should be at any rate a two-stage pressure drop to exhibit the development of hydrate.

The mechanism of hydrate formation can be described through a *P*–*t* curve as with three phases: dissolution, nucleation, and hydrate growth phase. The decrease in the initial pressure of the system, Po, from time (to) to time (ts) indicates the dissolution phase of hydrate formation. Cluster growth during hydrate formation occurs wherein the labile cluster will form immediately upon dissolution of gas in the water, and there are several types of clusters such as CO_2_ [(H_2_O)_2_O], CO_2_ [(H_2_O)_24_], and CO_2_ [(H_2_O)_28_]. Later, the pressure will become constant from time ts to time tr indicating the nucleation phase of hydrate formation with the total time from to to tr known as the induction time. At this stage, labile clusters will agglomerate to form dodecahedral, tetrakaidecahedral, or hexakaidecahedral clusters. Induction time usually refers to the time required to form the first clathrate hydrate cluster on which the microscopic hydrate grows.

Finally, when the size reaches a critical value, growth begins. The hydrate growth phase is represented by the curve from time tr until time td where the total time to achieve equilibrium can be obtained accordingly. Different SDS concentrations where several stages of pressure drop can be observed for each SDS concentration are obtained. Thus, this will be a basic guideline to determine the formation of CO_2_ hydrate in this work together with the study on CO_2_ dissolution in water. The equilibrium mole fraction of CO_2_ in water in the presence of hydrate at various operating temperatures and pressures is obtained.^40^ Thus, the use of T1-5 (5.60 mol % THF and 0.01 mol % SDS) combined promoters in the FBR which thermodynamically moved the hydrate phase equilibrium of the fuel gas mixture to the IGCC conditions within the sight of silica gel was relied upon because of the thermodynamic impact of TBAB and THF individually.^41^

### 3.9 Equilibrium of the Variable-Converted Hydrate Phase in IGCC Conditions

**Figure 9:**
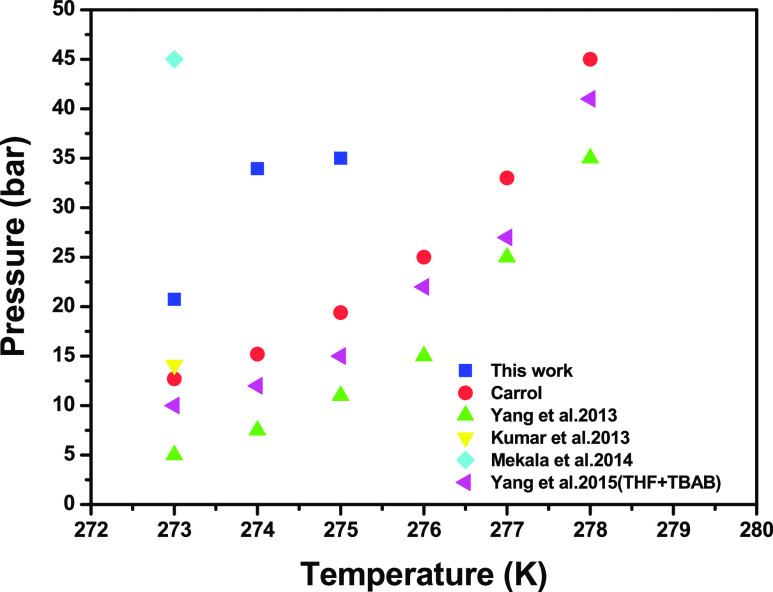
Hydrate phase equilibrium for pure CO_2_ gas of current work and contrasted with previous literature.

This research was the limit of combinations to move the phases of hydrate equilibrium to get higher performance. However, the final attempt of this isochoric framework has been used as a guideline to calculate the equilibrium pressure at test temperatures since most of the test pressures are set after hydrate development by 1200 min. The consumption of strong improvement will prompt the use of FBR, while STR is needed for a bulk system. Previously, relative work on the production of FBR by using CO_2_ gas was performed. Hydrate phase equilibrium of pure CO_2_ gas is shown in Figure 9. Furthermore, it has been accounted that SDS supplementation did not decrease the CO_2_–TBAB–H_2_O phase equilibrium while indicating that SDS is known as an extra dynamic fixing that can adjust the dynamic properties and not influence the hydrate phase equilibrium.

Appropriately, the assurance of 5.6 mol % THF and 0.1 mol % TBAB in T1-5 (5.60 mol % THF and 0.01 mol % SDS) and T3-2 (0.01 mol % SDS and 0.10 mol % TBAB) fused added substances unconventionally inside silica gel extended. Correlation of gas uptake at different IGGC working conditions and bed heights of T1-5 and T3-2 at a temperature of 283 K and pressure factors of 58 and 70 bar is mentioned in Table 3. Concerning the fuel gas framework, a couple of scientists who used the FBR and STR found that the phase equilibrium was moved to the higher temperature area as the atom size of silica gel extended from 6 to 100 nm. In any case, they reasoned that the utilization of silica gel in FBR moved the hydrate equilibrium phase to the limit region of bulk water because of the presence of geometrical constraints (slender effect).

**Figure 10:**
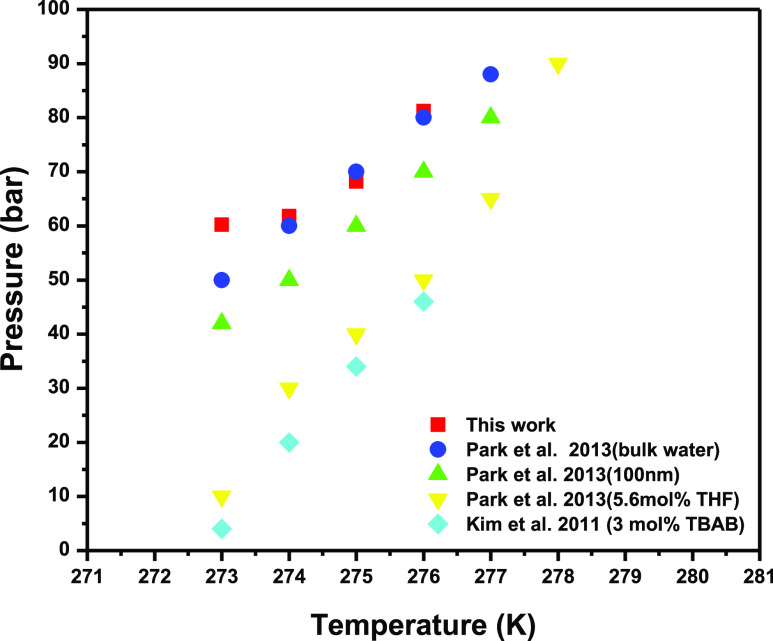
Hydrate phase equilibrium of fuel gas of this work and compared with previous literature.

The standard phase estimation of the THF–SDS–CO_2_–N_2_–H_2_O structure at the SDS fixed circumstance of 1000 ppm and the individual THF channels inside the presence of glass holders and the phase condition moved to a higher temperature. This has shown that the presence of THF can abnormally expand the main system solutions that also suggest relief of the hydrate setting. The formation of hydrate can also be obtained by TBANO_3_–CO_2_–H_2_–H_2_O system where TBANO_3_ is otherwise called as one kind of semiclathrate hydrate such as TBAB. It is found that by presenting TBANO_3_ in a fuel gas combination system, the heat of separation increased essentially, and this semiclathrate hydrate is supposed to be steadier than the hydrate formed from the fuel gas mixture.^39^ Hence, these contribute to moving the hydrate phase equilibrium to the higher temperature district wherein this effect is expected if TBANO_3_ is substituted by TBAB in that system. Figure 10 shows the hydrate phase equilibrium of fuel gas of this work, and it is compared with the literature. Recently, it has been figured out how to notice CO_2_ hydrate formation in the fuel gas mixture at the working temperature of 279–287 K and working pressure of 40–60 bar by utilizing 5.56 mol % THF in batch STR. For instance, CO_2_ hydrate formation at 285 K and 60 bar by utilizing silica sand saturated with 5.6 mol % THF inside FBR is feasible in the IGCC working conditions as shown in Figure 10.

## 4 Conclusions

In this work, tests for CO_2_ hydrate improvement inside the presence of SDS as a surfactant with EGME and THF as promoters were thought that EGME has been perceived as a choice as opposed to THF where the formation of CO_2_ hydrate (5.45 mmol of CO_2_/g of H_2_O) is refined by a molar concentration of 0.10 mol % together with 0.01 mol % SDS (picked type T1A-2). In any case, EGME at 1.00 mol % was found to forestall the development of CO_2_ hydrate which improves the double impact of EGME as both a promoter and an inhibitor in the lower and upper points individually. In this manner, more investigations on atomic demonstration are accepted, which can uncover the synergic impact of consolidating EGME and SDS with the goal that EGME can substitute THF as the promoter for CO_2_ hydrate formation. The inhibitory impact that appeared by silica gel accomplished with TBAB at 0.29 mol % was upgraded when blended with 0.01 mol % SDS where CO_2_ assimilation acquired by this T3-1 sample was improved. What is more, when TBAB spinal inclusion was diminished to 0.1 mol % and blended with 0.01 mol % SDS, the CO_2_ consumption of this T3-2 model was essentially improved which was 38% greater than the baseline experiment. However, the mixture of TBAB (0.29 mol %) and THF (3 mol %) was additionally inhibited by hydrate development when this T2-1 sample got 31% lower gas than TBAB (0.29 mol %) alone because of the combination of TBAB semiclathrate and THF hydrate. Further study on the selectivity of CO_2_ molecules toward hydrate formation in the fuel gas mixture by gas chromatography analysis and the improvement of reactor configuration by employing macroporous or mesoporous silica (silica sand or gel) with combined promoters is suggested for future work.
